# A Rare Case of Hepatic Sarcoidosis Caused By Hepatitis B Virus and Treatment-Induced Opportunistic Infection

**DOI:** 10.7759/cureus.10454

**Published:** 2020-09-14

**Authors:** Saumil Patel, Pinakin Patel, Rucha Jiyani, Sudeshna Ghosh, Divyank Patel

**Affiliations:** 1 Anesthesiology, M P Shah Medical College, Jamnagar, IND; 2 Gastroenterology, Bombay Hospital and Medical Research Centre, Mumbai, IND; 3 Internal Medicine, Nassau University Medical Center, East Meadow, USA; 4 Biochemistry, Institute of Post Graduate Medical Education & Research, Kolkata, IND; 5 Internal Medicine, Texas Tech University Health Sciences Center, El Paso, USA

**Keywords:** hbv, sarcoidosis, histoplasmosis, elastography, non caseating granulomas, langerhans cell

## Abstract

The association between hepatitis C virus (HCV) and sarcoidosis is well-documented, but in this case report, we shall discuss an interesting association between hepatitis B virus (HBV) and sarcoidosis, presenting with non-specific symptoms and confirmed with liver biopsy and immunologic markers. The case was complicated by treatment with immunosuppressive medication that led to colonic histoplasmosis.

A 58-year-old woman, from the western part of India, who has a past medical history of HBV-related cirrhosis of the liver for six months, hypertension, and type 2 diabetes presented to our clinic with bilateral pedal edema, anorexia, and mild epigastric discomfort. She had been on entecavir for the last six months. The patient denied any significant surgical, social, or family history. Abdominal ultrasonography revealed hepatosplenomegaly and mesenteric lymphadenopathy. She had a 21.3kPa liver stiffness on elastography and an HBV deoxyribonucleic acid (DNA) level of 89 copies/ml. Liver biopsy showed multiple noncaseating granulomas consisting of Langerhans cells in the parenchyma and portal tract, associated with moderate inflammation. A chest computed tomography (CT) scan showed upper and middle lobe fibrosis of the lungs; this diagnosis was further confirmed with elevated angiotensin-converting enzymes. She was started on prednisone; within a period of three months, she experienced weight loss, diarrhea, and fever. Colonoscopy was done after an abdomen CT showed mural thickening of the ascending colon and terminal ileum, which on biopsy was confirmed as histoplasmosis. Prednisone was stopped, and the patient was treated with hydroxychloroquine and amphotericin B, followed by itraconazole. The patient improved symptomatically, and repeated colonoscopy findings were normal.

Studies are scarce to prove the association between hepatitis B and sarcoidosis; however, we reasonably hypothesized that the alterations in the pool of cytokines and immune cells caused by HBV infection might have had a vicious influence on immune regulation and could be a trigger for granuloma. Further studies can impact the future to provide for a better understanding of the pathophysiology of sarcoidosis, HBV correlation, and treatment options.

## Introduction

The liver is involved in most patients who have sarcoidosis; among them, 50% to 65% have granulomas on liver biopsy, but symptomatic hepatic sarcoidosis occurs in 5% to 15% [1]. There have been documented cases reflecting the association between hepatitis C virus (HCV) and sarcoidosis, but here, we discuss the association of hepatitis B virus (HBV) and sarcoidosis in a patient presenting with non-specific symptoms, which is very rare. However, the diagnosis was confirmed with liver biopsy and immunologic markers. Of further interest is that treatment was complicated by immunosuppressive medication that led to colonic histoplasmosis.

## Case presentation

A 58-year-old woman with a known cause of hepatitis B virus (HBV)-related compensated cirrhosis of the liver for six months, type 2 diabetes, and hypertension for five years came to the hospital with the chief complaint of loss of appetite, abdominal bloating and discomfort, and bilateral pedal edema for one month but no weight change. She had a past medical history of an episode of jaundice 22 years prior (treated conservatively) and tubal ligation 25 years prior. The woman denied a history of hematemesis, melena, loss of consciousness, distension of the abdomen, or blood transfusion. There was no family history of cirrhosis of the liver or hepatocellular carcinoma. On examination, bilateral pitting edema up to the ankle was present. An abdominal examination revealed mild tenderness in the right hypochondrium with a palpable liver just below the right costal margin.

**Figure 1:**
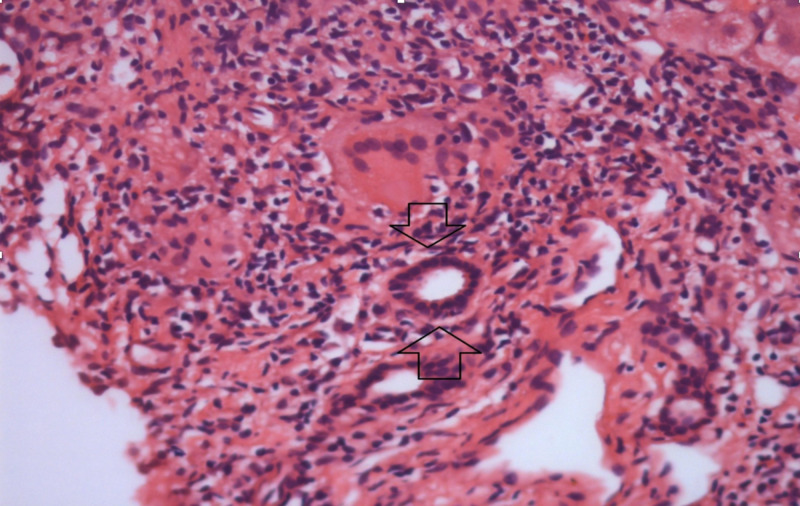
Moderate inflammation consists of lymphocytes and plasma cells in the portal area with mild interface activity and multiple small non-caseating granulomas in the portal tract and liver parenchyma

**Figure 2:**
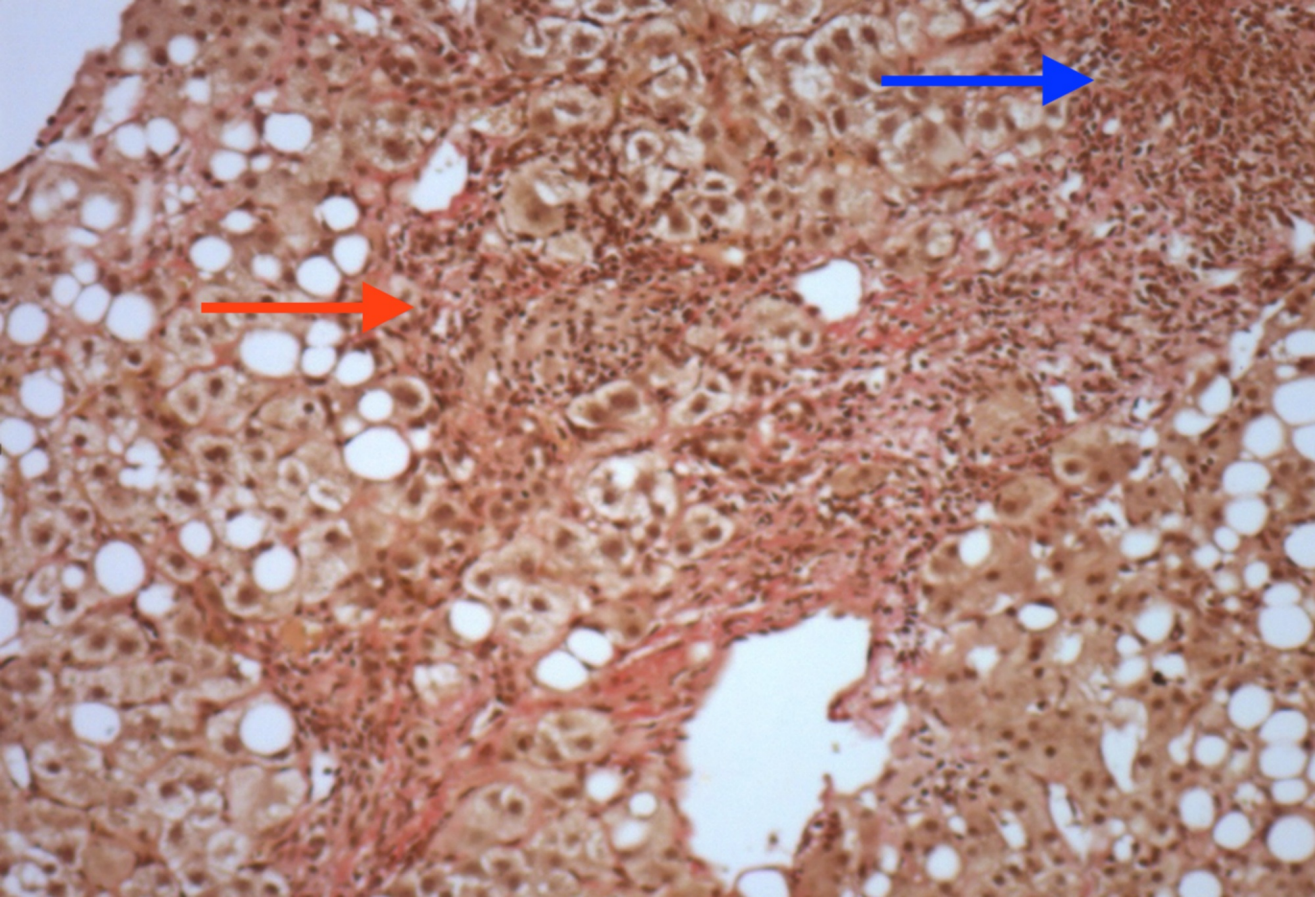
Macro-vesicular steatosis involving approximately 50% hepatocytes

An ultrasound (image record not available, now) of the abdomen showed diffuse coarse parenchymal echogenicity of the liver with hepatomegaly (14 cm), mild splenomegaly (12.6 cm), multiple sub-centimeter sizes mesenteric lymph nodes, and no ascites. The mean liver stiffness by transient elastography was 21.3 kPa. Esophagogastroduodenoscopy showed mild portal hypertensive gastropathy with no esophageal varices. Although she had been stable with chronic hepatitis B infection with a model for end-stage liver disease (MELD) score of 14, the Child-Pugh score was class A, the ultrasound showed hepatosplenomegaly with multiple mesenteric lymph and baseline HBV DNA levels at 15.89 IU/ml, so a liver biopsy (Figures 1-2) was performed, which revealed moderate inflammation consisting of lymphocytes and plasma cells in the portal area with mild interface activity and multiple small non-caseating granulomas, which consist of Langerhans giant cells in the portal tract and liver parenchyma. The Grocott-Gomori's Methenamine Silver (GMS) stain was negative for fungal infection.

A high-resolution CT (HRCT) (record not available now) of the chest showed fibrotic changes in the right upper and middle lobes with no mediastinal lymphadenopathy. Two sputum analysis was negative for acid-fast bacilli so tuberculosis was ruled out. Based on laboratory findings (Table 1), autoimmune hepatitis was ruled out because of the normal immunological marker. By correlating the biopsy finding of chronic hepatitis with cirrhosis, small non-caseating granulomas with an elevated angiotensin-converting enzyme level of 85.8 nmol/mL/min (reference range: less than 40 nmol/mL/min), and HRCT changes, hepatic sarcoidosis was diagnosed in this patient. But we also considered non-alcoholic steatohepatitis (NASH) as a differential diagnosis.

**Table 1:** Laboratory test results ACE, angiotensin-converting enzyme; ALP, alkaline phosphatase; ANA, antinuclear antibody; cmm, cubic millimeter; ESR, erythrocyte sedimentation rate; GGT, gamma-glutamyl transferase; HBV, hepatitis B virus; HCV, hepatitis C virus; MCV, mean corpuscular volume; PT INR, prothrombin time international normalized ratio; RA, rheumatoid arthritis; AST, aspartate aminotransferase; ALT, alanine aminotransaminase. fl: femtoliter, ASMA: anti-smooth muscle antibodies

Analyte	Result
Hemoglobin	10.5 g/dl
MCV	84.3 fl
Total count	6000/cmm
Platelet count	141,000/cmm
Total proteins	6.9 g/dl
Serum albumin	3.0 g/dl
Serum globulin	3.9 g/dl
HBsAg	positive
HBeAg	negative
Anti HBe antibody	positive
HBV DNA levels	< 3.8 IU/ml
Anti HCV	negative
HIV 1 & 2	negative
Serum IgG levels	2,119 mg/dl (normal up to 160)
Total bilirubin	2 g/dl
Direct bilirubin	1.2 g/dl
ALT	21 (15-63 mU/ml)
AST	46 (15-37 mU/ml)
ALP	222 (46-116 mU/ml)
GGT	109 (5-85 mU/ml)
INR	1.37
Serum creatinine	0.9 g/dl
Serum sodium	136 meq/dl
Serum B12	700 pg/ml
Serum D3	21.8 mg/ml
ESR	48 mm/hr
ACE level	85.8 nmol/mL/min (less than 40 nmol/mL/min)
ANA	negative,
RA and ASMA	negative

Based on these findings, we started the patient on oral prednisone at a dose of 40 mg/day for one month with ongoing oral entecavir 0.5 mg (which started at the baseline HBV DNA level of 15.89 IU/ml six months prior). The patient was better at follow-up visits, with improved appetite and no abdominal complaints, and liver function tests along with ACE level were also improved. So, we ruled out nonalcoholic steatohepatitis (NASH). Then, at subsequent visits, prednisone was tapered and then gradually tapered to 5 mg every week till 10 mg/day. After three months of treatment, she had developed a persistent low-grade fever with loose stools for 15 days, stool frequency of four to five times/day, which was watery, small in amount, without blood or mucus in stool, and associated with periumbilical abdominal pain. The patient had also lost 4 kgs of weight in the past month.

**Figure 3:**
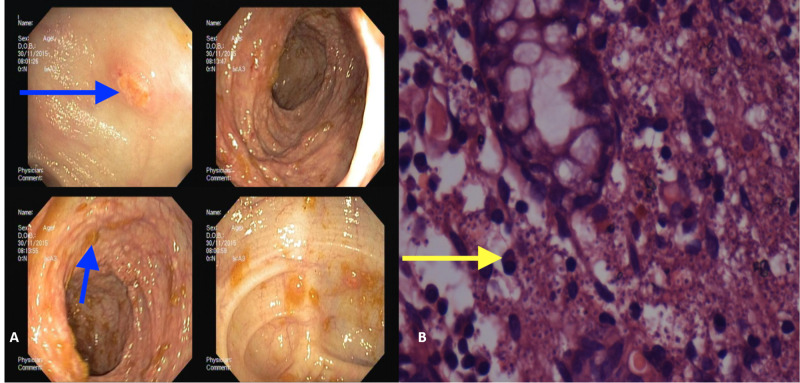
(A) Colonoscopy revealed multiple scattered ulcers (1–4 cm in diameter) throughout the colon; (B) Biopsy revealed lymphohistiocytic infiltrate with yeast forms (2-5 µm) with basophilic crescent-shaped nucleus seen within macrophages, often with a pericellular halo, most easily found within necrotizing granulomas

Her abdomen CT (record cannot be retrieved) showed hepato-splenomegaly with multiple mesenteric lymphadenopathies and mild, concentric, enhancing mural thickening involving the terminal ileum, ileocecal junction, and ascending colon. Colonoscopy (Figure 3A) was done and revealed multiple scattered ulcers (1-4 cm in diameter) throughout the colon. Biopsy (Figure 3B) was taken, which revealed, lymphohistiocytic infiltrate with yeast forms (2-5 µm) with basophilic crescent-shaped nucleus seen within macrophages, often with a pericellular halo, most easily found within necrotizing granulomas.

She was started on injection amphotericin B according to weight and continued for 21 days with renal function monitoring. After that, she was shifted to tablet itraconazole 200 mg for six weeks. Because of active colonic histoplasmosis, hydroxychloroquine was started for liver sarcoidosis and tablet prednisolone was stopped. At a follow-up after six weeks, she was clinically asymptomatic with normal colonoscopy and normal laboratory findings.

## Discussion

Worldwide, the prevalence of sarcoidosis varies from 20-60 per 100,000 people. In India, sarcoidosis constituted 61.2 per 100,000 new cases at regional institutes. In the United States, the lifetime risk of sarcoidosis in black patients is 2.4% as compared with a lifetime risk of 0.85 percent in white patients. In India, the prevalence of HBV is 2% to 7%, with an average of 4%, with a burden of approximately 50 million people with chronicity [2-6].

There are a few cases reported that suggest an association between sarcoidosis and HCV infection, which is mostly due to the treatment of interferon-alpha (IFN-α) in HCV infected patients. The association between sarcoidosis and HBV is very rare. In this case, a patient with chronic HBV infection developed hepatic sarcoidosis but had never been treated by IFN-α. There are reported cases of IFN-α induced hepatic sarcoidosis in chronic hepatitis virus infection. Thus, we believed there might be a possible clinical correlation between hepatic sarcoidosis and HBV infection rather than they co-existed independently, though relevant studies are rare.

We suspect that the different mechanisms of production of cytokines in HBV and HCV infection might induce sarcoidosis. During the pathogenesis of sarcoidosis, it is noted that there is a high expression of T-helper 1 cells that produce cytokines, such as IFN-γ and interleukin-2, and IFN-γ is also secreted in HBV infection. Though the exact mechanism remains unclear, it can be reasonably assumed that the dysregulation of cytokines and immune cells caused by HBV infection can be a trigger for granuloma formation [7-11].

In addition to that, our patient had colonic histoplasmosis on steroid treatment for sarcoidosis [12]. It is well-known that immune-suppressive conditions predispose for histoplasmosis. The depression of cell-mediated immunity occurs on steroid treatment and has been attributed to shortened lymphocyte survival, lymphopenia, inhibition of lymphocyte transformation, and suppressor T cell activity. This cellular immune deficiency may have been the risk factor in our patient, predisposing her to infection from Histoplasma capsulatum. So, it further challenges sarcoidosis treatment.

## Conclusions

This report describes a rare case of hepatic sarcoidosis accompanied by liver cirrhosis in a patient with chronic HBV infection. Interestingly, he had never been treated with IFN-α, the usual cause of sarcoidosis in a patient with hepatitis virus infection. Further studies should be conducted to explore the potential causal relationship between hepatitis B virus infection and the development of sarcoidosis. The use of immune-suppressive treatment for sarcoidosis presents a challenge because it would increase the risk of opportunistic infection.
